# Central Vein Sign and Paramagnetic Rim Lesions in Patients with Relapsing–Remitting Multiple Sclerosis: An Assessment of Prevalence and Anatomical Location

**DOI:** 10.3390/neurolint18050095

**Published:** 2026-05-20

**Authors:** Marija Nikola Jansone, Nauris Zdanovskis, Elina Polunosika, Daina Pastare, Guntis Karelis

**Affiliations:** 1 Department of Radiology, Riga Stradiņš University, Dzirciema iela 16, LV-1007 Riga, Latvia; marijanikola.jansone@rsu.edu.lv; 2 Department of Radiology, Riga East University Hospital, Hipokrata iela 2, LV-1038 Riga, Latvia; 3 Institute of Public Health, Riga Stradiņš University, Dzirciema iela 16, LV-1007 Riga, Latvia; 4 Department of Neurology and Neurosurgery, Riga East University Hospital, Hipokrata iela 2, LV-1038 Riga, Latvia; elinapolunosika@gmail.com (E.P.); daina.pastare@rsu.lv (D.P.); guntis.karelis@rsu.lv (G.K.); 5 Department of Neurology and Neurosurgery, Riga Stradiņš University, Dzirciema iela 16, LV-1007 Riga, Latvia; 6 Department of Infectology, Rīga Stradiņš University, Dzirciema iela 16, LV-1007 Riga, Latvia

**Keywords:** central vein sign, paramagnetic rim lesions, multiple sclerosis, white matter lesions, McDonald criteria, susceptibility-weighted angiography

## Abstract

Background/Objectives: Multiple sclerosis (MS) remains challenging to diagnose due to clinical and radiological overlap with mimicking conditions. The 2024 revisions of the McDonald criteria have incorporated the central vein sign (CVS) and paramagnetic rim lesions (PRLs) as magnetic resonance imaging (MRI) biomarkers to improve diagnostic specificity. This study assessed the prevalence and anatomical distribution of CVS and PRLs in patients with relapsing–remitting MS (RRMS). Methods: This cross-sectional study included 91 patients with RRMS diagnosed according to the 2017 McDonald criteria. MRI scans were obtained using 3T scanners, and T2-FLAIR and susceptibility-weighted angiography (SWAN) sequences were analyzed. CVS and PRLs were identified using established criteria. Patients were stratified by lesion count (<5, 5–9, ≥10), and lesions were categorized by anatomical location. Descriptive statistics, chi-square tests, and multivariable logistic regression adjusted for covariates were performed. Results: CVS was present in 69.2% of patients, while PRLs were identified in 29.7%. Both markers were more frequent in patients with higher lesion burden in univariate analysis. CVS prevalence increased significantly with lesion count (*p* < 0.001) and remained an independent predictor in multivariable logistic regression. PRL presence was associated with lesion count in univariate analysis but not after adjustment. Most CVS- and PRL-positive lesions were supratentorial and predominantly periventricular. No significant association was observed between CVS and PRL presence. Conclusions: CVS is a highly prevalent MRI feature in RRMS and independently associated with lesion burden, supporting its role as a diagnostically relevant imaging marker. PRLs were less prevalent and showed weaker independent associations.

## 1. Introduction

Multiple sclerosis (MS) misdiagnosis remains a contemporary problem due to the clinical and radiological overlap with several disorders, influencing patient outcomes. Recent cohort studies suggest that up to 18% of patients were found to have been misdiagnosed with MS [1]. One of the primary reasons for MS misdiagnosis is the misinterpretation of or overreliance on magnetic resonance imaging (MRI) abnormalities in the setting of nonspecific neurologic symptoms [2]. Recent efforts have focused on the validation of new imaging biomarkers to enhance the diagnostic accuracy of multiple sclerosis [3]. This is reflected in the 2024 revisions of the McDonald MS diagnostic criteria, which have incorporated the central vein sign (CVS) and paramagnetic rim lesions (PRLs) as imaging biomarkers to improve diagnostic specificity, highlighting their increasing relevance in clinical practice [4,5].

CVS is characterized by a small hypointense line or dot running centrally through a white matter lesion (WML) on susceptibility-weighted imaging (SWI) or susceptibility-weighted angiography (SWAN) sequences [6]. Studies indicate that the use of CVS substantially improves diagnostic specificity [7]. While 7T MRI provides the highest sensitivity for detecting CVS due to its superior spatial resolution, 3T MRI remains the most widely used modality in clinical settings [8] and has proven effective for identifying CVS. At 3T, 93.4% sensitivity and 100% specificity have been reported using a >12% threshold of lesions with CVS [9].

Another emerging imaging biomarker is the PRL, which represents a chronic active demyelinating lesion with iron-laden microglia at its edge. PRLs appear as a hypointense rim on SWI or SWAN around WMLs [10,11]. PRLs are notably less common than lesions exhibiting the CVS. In a 7T MRI study of MS patients, only 13.1% of white matter lesions exhibited a paramagnetic rim, compared to 82.2% showing the CVS, highlighting the lower prevalence of PRLs relative to CVS-positive lesions [12]. Due to their relatively low prevalence, PRLs demonstrate lower sensitivity as a diagnostic marker for MS, although their specificity remains high. The detection of at least one PRL was shown to have a sensitivity of 24.0% and a specificity of 99.7% in distinguishing MS and clinically isolated syndrome from non-MS controls [13].

As CVS and PRLs are incorporated into the 2024 revisions of the McDonald diagnostic criteria, examining the presence of these biomarkers in a cross-sectional sample of diagnosed MS patients provides insight into their prevalence and characteristics. The primary objective of this study was to determine the prevalence of CVS and PRLs in patients with relapsing–remitting MS (RRMS) and to evaluate their association with lesion burden. The secondary objective was to describe the anatomical distribution of these imaging markers. We hypothesized that patients with a higher lesion burden would exhibit a greater likelihood of both CVS and PRL presence.

## 2. Materials and Methods

### 2.1. Participants

In this cross-sectional study, 91 patients with RRMS were recruited at the Riga East University Hospital. All participants met the 2017 McDonald criteria for RRMS diagnosis [14], were between 21 and 64 years old, and had a disease duration of no more than 20 years. Exclusion criteria were insufficient SWAN and/or FLAIR (fluid-attenuated inversion recovery) image quality, such as motion artifacts, which could affect imaging analysis. Neurological disability was assessed using the Expanded Disability Status Scale (EDSS), a validated and widely used clinical scale developed by Kurtzke to quantify disability in multiple sclerosis patients [15]. EDSS scoring was performed by a trained neurologist as part of routine clinical evaluation. No modifications to the scale were made for the purposes of this study.

### 2.2. MRI Acquisition

MRI examinations were performed on 3T scanners using harmonized acquisition protocols. Sequence design, spatial resolution, and contrast weighting were standardized across scanners to minimize inter-scanner variability. The protocol included SWAN-venule imaging (FOV 22 × 16 cm; 126 slices; voxel size 0.4 × 0.4 × 0.8 mm, reconstructed to 0.4 mm isotropic; TR 47 ms; TE 28 ms; flip angle 8°; ETL 9; acquisition time 7:38 min) and 3D T2-weighted FLAIR (FOV 26 × 26 cm; 146 slices; voxel size 0.47 × 0.47 × 1.2 mm; TR 6200 ms; TE 116 ms; inversion time 1710 ms; ETL 220; acquisition time 4:18 min). Where minor scanner-dependent deviations were unavoidable, they were limited through protocol harmonization.

### 2.3. Image Analysis

**Figure 1:**
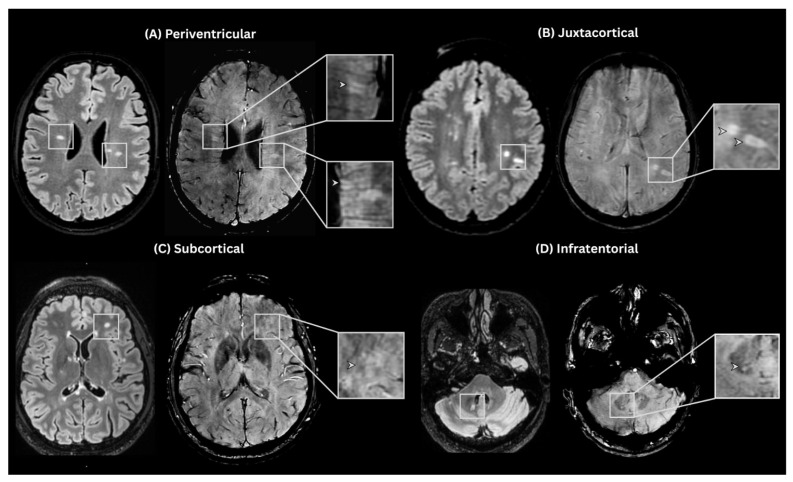
Examples of CVS-positive white matter lesions across four anatomical locations. Representative paired T2-FLAIR and SWAN images demonstrate CVS-positive lesions. On FLAIR, lesions appear as hyperintense foci, while the corresponding SWAN images reveal a central intralesional vein.

MRI image analysis was performed using T2-FLAIR and SWAN sequences. White matter lesions were assessed on T2-FLAIR sequence, and patients were grouped based on the total number of lesions across all typical MS locations into categories: <5, 5–9, and ≥10 lesions. The presence of CVS was assessed on SWAN in accordance with the North American Imaging in Multiple Sclerosis (NAIMS) guidelines [6]: a small hypointense line or dot, less than 2 mm in diameter, running centrally through a focal lesion larger than 3 mm, and visible in at least two perpendicular MRI planes (Figure 1).

**Figure 2:**
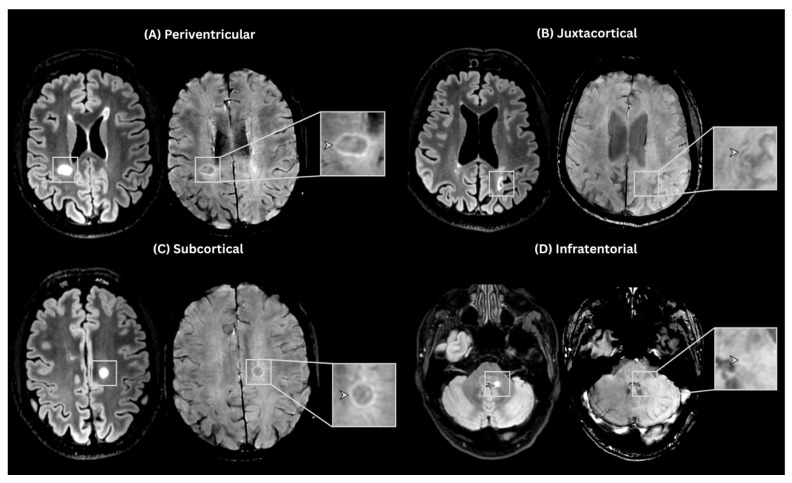
Examples of PRLs across different anatomical locations. Representative paired T2-FLAIR and SWAN images demonstrate PRLs. On FLAIR, lesions appear as hyperintense foci, while the corresponding SWAN images show a hypointense paramagnetic rim.

A PRL was assessed on SWAN and defined following the NAIMS consensus statement [3]: discrete, continuous rim with paramagnetic properties on susceptibility-sensitive MRI sequences, visible on at least two consecutive slices, covering at least two-thirds of the lesion’s white matter edge. (Figure 2) Lesions with CVS or PRL were categorized based on anatomical location—periventricular, subcortical, juxtacortical and infratentorial.

All MRI scans were evaluated by a board-certified radiologist with experience in neuroimaging. Lesion assessment was performed manually based on established criteria for CVS and PRL identification as described above. Readings were performed once without formal blinding.

### 2.4. Statistical Analysis

Statistical analysis was performed using JASP (version 0.19.3; Amsterdam, The Netherlands) and *p*-values < 0.05 were considered significant. The analysis included descriptive statistics, the chi-square test, and multivariable logistic regression. Descriptive statistics were calculated to summarize participant characteristics, lesion distribution, and the prevalence of CVS and PRL. Associations between categorical variables were assessed using the Pearson chi-square test. Multivariable logistic regression evaluated whether lesion count group predicted CVS or PRL presence after adjustment for age, sex, disease duration, and EDSS, with model performance reported using Nagelkerke R^2^ and area under the curve (AUC).

## 3. Results

### 3.1. Patient Demographics

This study included 91 patients with relapsing-remitting multiple sclerosis. Among the 91 patients were 57 female (63%) and 34 male (37%). Sex was recorded as biological sex (female/male) based on medical records. Mean age was 41.6 years. The median (range) EDSS score was 2.0 (1–6.5), indicating mild to moderate disability, and median (range) disease duration was 6 (1–20) years. Based on lesion count, 13.19% (12/91) of patients had fewer than five lesions, 20.88% (19/91) had 5–9 lesions, and 65.93% (60/91) had more than ten lesions (Table 1).

### 3.2. CVS Analysis

**Figure 3:**
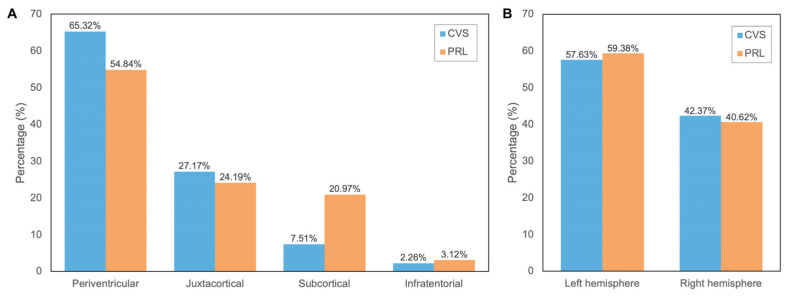
Anatomical (**A**) and hemispheric (**B**) distribution of CVS and PRLs.

CVS was present in 69.23% (63/91) of patients. The prevalence of CVS increased markedly with lesion burden. Only 4.67% (3/63) of patients with fewer than five lesions demonstrated CVS, compared with 14.29% (9/63) in the 5–9 group and 80.95% (50/63) in the ≥10 lesion group. Percentages are calculated among patients with at least one CVS-positive lesion. A total of 177 CVS-positive lesions were identified. These showed a slight left-hemispheric predominance 57.63% (102/177), compared to the right hemisphere 42.37% (75/177). Most CVS-positive lesions were located in supratentorial regions 97.74% (173/177), with only 2.26% (4/177) found infratentorially. Within the supratentorial compartment, periventricular lesions accounted for the majority 65.32% (113/173), followed by juxtacortical 27.17% (47/173) and subcortical 7.51% (13/173) regions (Figure 3).

A chi-square test demonstrated a significant association between CVS presence and lesion count group (χ^2^ = 22.288, df = 2, *p* < 0.001). Multivariable logistic regression was then performed to determine whether this association remained significant after adjusting for age, sex, disease duration, and EDSS. In the adjusted model, having more than ten lesions was the only independent predictor of CVS presence (OR 7.8, 95% CI 2.18–27.8, *p* = 0.002). Neither age, sex, disease duration, nor EDSS contributed significantly to the model. Overall model fit was robust (Δχ^2^ = 24.211, *p* < 0.001), explaining 32.9% of the variance in CVS presence (Nagelkerke R^2^ = 0.329). The receiver operating characteristic (ROC) curve analysis demonstrated good discriminative ability with an AUC of 0.789 (Table 2).

### 3.3. PRL Analysis

PRLs were identified in 29.67% (27/91) of patients. Similarly to CVS, a higher PRL prevalence was observed in patients with higher lesion counts. At least one PRL was found in 85.19% (23/27) of patients with lesion count ≥ 10, 11.11% (3/27) of patients with lesion count 5–9, and 3.7% (1/27) with lesion count < 5. PRLs were more frequently observed in the left hemisphere 59.38% (38/64) compared to the right hemisphere 40.62% (26/64). PRLs exhibited a predominantly supratentorial distribution 96.88% (62/64), with periventricular regions comprising 54.84% (34/62) of PRL-positive lesions, followed by juxtacortical at 24.19% (15/62) and subcortical areas at 20.97% (13/62). Infratentorial PRLs were rare at 3.12% (2/64) (Figure 3).

A chi-square test showed a significant association between lesion count group and PRL presence (χ^2^ = 6.530, *p* = 0.038). However, in contrast to CVS, this relationship did not remain significant in the adjusted logistic regression model (OR 2.33, 95% CI 0.57–9.53, *p* = 0.238). No demographic or clinical covariates reached significance, though disease duration showed a non-significant trend toward higher PRL likelihood (*p* = 0.052). The model accounted for 21.4% of variance (Nagelkerke R^2^ = 0.214) with acceptable discriminative performance (AUC = 0.731) (Table 2).

The relationship between CVS presence and PRL presence was also analyzed. A chi-square test indicated no significant association between the two biomarkers (χ^2^ = 2.705, df = 1, *p* = 0.100). Logistic regression likewise indicated that having CVS-positive lesions did not predict PRL occurrence (OR 2.47, *p* = 0.106; Nagelkerke R^2^ = 0.044).

## 4. Discussion

In this cross-sectional study, we evaluated the prevalence and anatomical distribution of the CVS and PRLs on 3T SWAN images in a cohort of 91 patients diagnosed with RRMS. Our findings revealed an overall patient-level prevalence of CVS in 69.23% of the cohort and PRLs in 29.67%. Both CVS and PRLs were more frequently observed in patients with higher lesion burden. The anatomical distribution was predominantly supratentorial. Within the supratentorial region, CVS-positive lesions and PRLs were most frequently located in the periventricular area, followed by juxtacortical and then subcortical regions.

The high prevalence of CVS observed in our RRMS cohort suggests that perivenular inflammation is a fundamental characteristic of MS pathology, even in its relapsing-remitting stage. This observation aligns with the well-established understanding that MS lesions exhibit a perivenular distribution, originating from inflammatory infiltrates around small veins [16]. Our findings regarding the prevalence of CVS at 3T MRI align with existing literature. A meta-analysis [17] reported a pooled proportion of 73% of MS lesions showing CVS, and a patient-level prevalence of 74% in studies using 3T MRI. Our observation of predominantly supratentorial and periventricular CVS-positive lesions is also supported by previous research. Sparacia et al. [18] reported that 55.5% of CVS-positive MS lesions at 3T were periventricular.

Regarding PRL prevalence, our patient-level finding of 29.67% is within the range reported in other 3T MRI studies. A meta-analysis by Ng Kee Kwong et al. [19] estimated a pooled patient-level prevalence of PRLs at 3T to be 24.4%. The predominantly periventricular location of PRLs in our cohort does not align with other studies. Other research suggests a higher prevalence in deep white matter [20,21]. This discrepancy in anatomical distribution might stem from variations in patient cohorts, MRI protocols, and the precise definition used for identifying PRLs.

The lack of a significant association between CVS and PRLs in our cohort is consistent with findings from studies suggesting that these biomarkers may reflect distinct pathological processes. These markers may reflect different stages of lesion development in MS—from initial inflammation to more chronic, active lesions that accumulate iron over time [22]. While our cross-sectional study cannot directly assess prognosis, the observed association of PRLs with higher lesion burden aligns with findings from longitudinal studies. These studies indicate that PRLs might serve as markers of a more aggressive disease course and potentially predict future disability progression [23,24].

The main limitation of our study is the absence of a non-MS control group, limiting evaluation of CVS and PRL specificity in distinguishing MS from mimicking conditions. The cross-sectional design prevents assessment of lesion progression, and only RRMS patients were included, reducing generalizability to progressive MS. Observer bias is possible, as lesion assessment was performed manually by a single radiologist, and the study lacks longitudinal progression data to evaluate how CVS and PRLs evolve over time. Additionally, patient selection and data collection were performed prior to the publication of the 2024 McDonald criteria.

Despite these limitations, our findings contribute to the growing body of evidence supporting the potential clinical utility of CVS and PRLs as valuable imaging biomarkers for MS. The relatively high prevalence of CVS in our RRMS cohort further reinforces its role in characterizing MS lesions. Future studies could also evaluate abbreviated CVS counting approaches, such as the Select-3 and Select-6 criteria, which may further improve the clinical applicability of CVS assessment [25]. The high specificity of PRLs, even with a lower prevalence, suggests their potential to enhance diagnostic confidence. Our results also support the feasibility of detecting these biomarkers using standard clinical 3T MRI protocols, highlighting their potential for widespread implementation. The incorporation of these markers into the 2024 McDonald diagnostic criteria for MS reflects the increasing recognition of their diagnostic value.

## 5. Conclusions

Our study provides further evidence on the prevalence and anatomical distribution of CVS and PRLs in patients with RRMS using 3T MRI. The high prevalence of CVS and the predominantly supratentorial and periventricular distribution of both biomarkers align with existing literature. The lower prevalence of PRLs and the lack of significant association between CVS and PRLs suggest that these markers may reflect different aspects of MS pathology. While our findings contribute to the growing understanding of these imaging biomarkers, future research, particularly longitudinal and multi-center studies, is needed to further validate their clinical utility and integrate them into routine diagnostic and prognostic assessments for multiple sclerosis.

## Figures and Tables

**Table 1:** Descriptive statistics of patient demographics and lesion count groups.

Characteristic	Value
Number of patients	91
Female, No./No. (%)	57/91 (63%)
Age, mean (SD) [Range], y	41.6 (10.4) [21.0–64.0]
Disease duration, median (Range), y	6 (1–20)
EDSS score, median (range)	2 (1–6.5)
Lesion count, No./No. (%)	
<5	12/91 (13.19%)
5–9	19/91 (20.88%)
≥10	60/91 (65.93%)

**Table 2:** Associations between clinical variables and the presence of CVS and PRL.

	CVS		PRL	
	*p*-Value	Odds Ratio	*p*-Value	Odds Ratio
Sex	0.167	0.449	0.150	0.672
Age	0.494	1.020	0.445	0.980
Disease duration	0.714	0.973	0.052	1.135
EDSS	0.912	0.972	0.240	1.287
Lesion count				
<5	0.294	0.414	0.430	0.367
≥10	0.002	7.793	0.238	2.332

## Data Availability

The data that support the findings in this study are available from the corresponding author upon request.

## Abbreviations

The following abbreviations are used in this manuscript:

AUC	Area under the curve
CVS	Central vein sign
EDSS	Expanded Disability Status Scale
MRI	Magnetic resonance imaging
MS	Multiple sclerosis
NAIMS	North American Imaging in Multiple Sclerosis
PRL	Paramagnetic rim lesion
ROC	Receiver operating characteristic
RRMS	Relapsing–remitting multiple sclerosis
SD	Standard deviation
SWAN	Susceptibility-weighted angiography
SWI	Susceptibility-weighted imaging
WML	White matter lesion
